# Age-related effects on dynamic postural stability and prefrontal cortex activation during precision fitting tasks

**DOI:** 10.7717/peerj.18548

**Published:** 2025-01-29

**Authors:** Jiahao Pan, Hui Tang

**Affiliations:** 1 Biomedical Engineering Doctoral Program, Boise State University, Boise, ID, United States of America; 2 Department of Kinesiology and Health Education, The University of Texas at Austin, Austin, TX, United States of America

**Keywords:** Goal-directed behavior, Postural control, Attentional demands, Neural dysfunction, Neural compensation

## Abstract

**Background:**

Dynamic postural control is impaired in older adults, as evidenced from worse dynamic postural stability compared to young adults during upright stance while concurrent goal-directed tasks. Prefrontal cortex (PFC) is considered to play an important role in goal-directed tasks. This study aimed to investigate the age effects on dynamic postural stability and PFC activation during precision fitting tasks.

**Methods:**

Participant performed precision fitting tasks under four different conditions: large opening size with their arm’s length (close-large), small opening size with their arm’s length (close-small), large opening size with 1.3 times arm’s length (far-large), and small opening size with 1.3 times arm’s length (far-small). We analyzed the center of pressure-related outcomes representing dynamic postural stability and PFC activation at the six different subregions from healthy older adults (*n* = 15, 68.0 ± 3.5 years), and gender-matched middle-aged (*n* = 15, 48.73 ± 3.06 years) and young (*n* = 15, 19.47 ± 0.64 years) adults.

**Results:**

The dynamic postural stability presented the young > middle-aged > older groups across the conditions. Specifically, the young group presented better dynamic postural stability than the older group in the close-large, far-large, and far-small conditions (*p* < .05), while showed better dynamic postural stability than the middle-aged group in the close-large condition (*p* < .05). Additionally, the older group had greater PFC activation at all PFC subregions than the young group (*p* < .05), while had greater activation at left dorsolateral and ventrolateral PFC than the middle-aged group (*p* < .05). The middle-aged group presented greater activation at left dorsomedial PFC than the young group (*p* < .05).

**Conclusion:**

Heightened dorsomedial PFC activation in middle-aged adults compared to young adults may reflect a deficit in processing the visuomotor information during the precision fitting tasks. Degeneration of the ability in automatic coordination of dynamic postural control may begin to occur at midlife.

## Introduction

The ability to adopt and adjust posture while maintaining an upright stance and performing concurrent goal-directed tasks, such as reaching, grasping, and fitting, is crucial for daily activities (Clark, Czaja & Weber, 1990; Tedeschi, 2023). Aging, however, is associated with a noticeable decline in this dynamic postural control (Bouisset & Do, 2008). With age, individuals tend to shift from a flexible to a stiffer, less adaptive posture during such tasks, posing challenges in mitigating perturbations and sustaining dynamic postural stability (Haddad et al., 2013). Older adults exhibit worse dynamic postural stability than young adults, evidenced by greater center of pressure (CoP) displacement and sway area, along with reduced CoP trajectory smoothness during the goal-directed tasks with upright stance (Huang & Brown, 2013; McNevin, Weir & Quinn, 2013). This deficit in dynamic postural control may indicate more fall risks in older populations (Haddad et al., 2013). Age-related loss of postural automaticity may be associated with the deficits in dynamic postural control (Clark, 2015). Older adults may allocate additional attention-demanding executive control resources to compensate for deficits in dynamic postural control (Clark, 2015). Therefore, understanding the neural processing behind this age-related dynamic postural instability is vital for developing effective interventions to enhance dynamic postural control and minimize fall risks for the elderly, particularly in complex, multitasking scenarios.

The prefrontal cortex (PFC) plays an important role in processing the goal-directed tasks (Funahashi & Andreau, 2013; Fuster & Bressler, 2015; Glover, Wall & Smith, 2012; Kaller et al., 2011; Mederos et al., 2021; Schöner, Bildheim & Zhang, 2024; Yamagata et al., 2012). In general, increased in PFC activation would linked to greater degree of attention-demanding executive control adjustment during the complexed goal-directed tasks (Mansouri, Tanaka & Buckley, 2009). It is because automatic or previously learned behaviors may no longer achieve the given task (Mansouri, Tanaka & Buckley, 2009). Different PFC sub-regions, like the dorsolateral (engaged in retrieving behavioral-goal information) and ventrolateral (involved in encoding object features) areas, would process different information during the goal-directed tasks (Yamagata et al., 2012). Also, the dorsomedial region of PFC is key in dynamically reconfiguring visuomotor-related functional connectivity networks, integrating sensory input and motor planning for precise coordination during the goal-directed tasks (Brovelli et al., 2017). Therefore, monitoring the PFC activation in the different subregions would reflect age-related change in ability to process different information during the goal-directed tasks.

Postural automaticity reflects the ability of coordinating dynamic postural control with minimal use of attention-demanding executive control resources (Anderson, 2018; Gaveau et al., 2014; Huang & Brown, 2015; Potocanac & Duysens, 2017). Prior MRI study reported that performing simple reaching and grasping movements did not engage the PFC activation compared to planning without executing them. This finding indicated the automatic nature (Glover, Wall & Smith, 2012). In such condition, individuals’ postural response could be rapidly and flexibly altered to adapt the concurrent arm movement (Galgon, Shewokis & Tucker, 2010; Huang & Brown, 2015; Leonard et al., 2011; Lowrey, Nashed & Scott, 2017). For instance, healthy young to middle-aged adults present better dynamic postural stability and higher hand accuracy in response to the goal-directed tasks after enough practice (Galgon, Shewokis & Tucker, 2010). However, the age-related deficits in generating appropriate postural response to achieve the optimized online movement trajectory was observed when performing the goal-directed tasks (Goodman et al., 2018; Goodman & Tremblay, 2021; Haaland, Harrington & Grice, 1993; Sarlegna, 2006). As aging advances, older adults need longer planning durations, exhibit worse dynamic postural stability, and rely more on sensory feedback and cognitive processing to compensate the planning deficiencies and to correct the online movement trajectory of the goal-directed tasks (Goodman & Tremblay, 2021; Huang & Brown, 2015; Sarlegna, 2006). Although these studies highlight the significant role of brain in processing the goal-directed tasks, they have not directly examined the altered physiological brain function, particularly in the PFC (Goodman & Tremblay, 2021; Huang & Brown, 2015). Therefore, there is a research question whether older adults greater PFC activation and worse dynamic postural stability compared to young and middle-aged adults in response to the goal-directed tasks during upright standing.

Throughout the lifespan, the high-order cognitive function and postural control system have shown gradual degeneration, starting in the midlife and continuing thereafter (Era et al., 2006; Peters, 2006). For example, middle-aged adults showed reliance more on attention-demanding executive control resources assessed by greater PFC activation compared to young adults during cognitive memory tasks (Klaassen et al., 2016; Kwon et al., 2016). In terms of postural control, a slight but noticeable decline in balance is observed in adults aged 40 to 49 compared to those 30 to 39, with significant deterioration after 60 years old (Era et al., 2006). It is reasonable to suggest that the degeneration of automatic coordination of dynamic postural control in goal-directed tasks may also commence in midlife.

This study investigated the age-related effect on the PFC activation and dynamic postural stability during the precision fitting tasks. We hypothesized that (1) older adults would present a higher PFC activation and worse dynamic postural stability than the middle-aged and young adults during the precision fitting task; and (2) middle-aged adults would also show a higher PFC activation than the young adults, but no significant difference in dynamic postural stability during the precision fitting task.

## Methods

### Participants

We recruited right-handed healthy young, middle-aged, and older adults in the current study. The inclusion criteria were as follows: (1) age 60 or older in the older group, age from 45 to 55 in the middle-aged group, age from 18 to 22 in the young group; (2) able to stand and walk at least 2 min without any assistance; (3) no injury or surgery at lower extremity in the past 6 months; (4) no neurological diseases, such as mild cognitive impairment, permanent memory loss, stroke, Parkinson’s disease, and brain tumors; (5) Mini-Mental State Examination (MMSE) score ≥ 24 for all three groups, except for participants with 0 to 6 years of schooling (MMSE score ≥ 22) (Cui et al., 2011); and (6) no history of drug and alcohol abuse. The authors have permission to use MMSE from the copyright holders. This study was approved by the Ethics Committee of the Affiliated Hospital of Yangzhou University (2020-YKL12-23-02). Each participant signed the informed consent form before participation.

### Instrumentation setup

A custom-built instrument was utilized in this study, featuring a large whiteboard with two openings: a larger one measuring 130 × 130 mm and a smaller one measuring 100 × 100 mm. These openings were positioned in the upper middle section of the whiteboard, spaced 150 mm apart. Additionally, a fitting block measuring 90 × 90 mm, equipped with a cylindrical handle (20 mm in length and 10 mm in diameter), was placed on a custom-built base situated on a small table. Both the fitting board and the small table were designed with adjustable heights and positions. Finally, four pairs of optical sensors were employed, which are synchronized with Vicon Nexus system (Version 2.5, Vicon, Inc., Oxford, UK). Of these, two pairs were affixed to the top middle edge of each opening and on the whiteboard’s backside, while the remaining pairs were attached to each side of the custom-built base.

The CoP data was collected using an embedded force plate (Kistler 9285BA, Kistler Corporation, Winterthur, Switzerland) at a sampling rate of 2,000 Hz. Data collection from the force plate was conducted using Vicon Nexus system (Version 2.5, Vicon, Inc., Oxford, UK). Changes of the cortical activation in the PFC were measured using an fNIRS device (Brite 24, Artinis medical systems, Einsteinweg, Netherlands) at a sampling rate of 50 Hz, utilizing two wavelengths of near-infrared light (760 and 850 nm). The setup included 10 sources and eight detectors, constituting 24 channels in total, positioned on the head’s surface *via* a standard NIRS cap (10–10 international system) covering the PFC. The differential pathlength factor (DPF) was calculated was age-dependent: for participants younger than 55 years, DPF was determined using the formular: 4.99 + 0.067 × Age^0.814^ (Duncan et al., 1996). For participants older than 55, the DPF was fixed set to 6.61 (Claassen, Levine & Zhang, 2009). To identify the subregions of PFC, five anatomical landmarks (nasion, inion, Cz, left and right preauricular points) were digitized using a Polhemus digitizer. Oxysoft was used for the collection prefrontal cortex activation. All devices were synchronized.

### Study protocol

This study was a cross-sectional design. Participants performed the precision fitting task in this study. The precision fitting task entails both the execution of typical goal-directed task and the need to maintain dynamic postural stability (Pan et al., 2020; Pan et al., 2016). Moreover, task constraints could be simply manipulated by decreasing the opening size (enhancing the fitting precision) and increasing the opening distance (enhancing the postural constraint) (Coats et al., 2016; Huang & Brown, 2013; Potocanac & Duysens, 2017; Sarlegna, 2006).

Participants wore uniform socks provided by the laboratory. Each participant stood on the center of the force plate with feet forming a 30° angle, heels being apart at 8% of the height, arms relax on each side, and align their middle line with the opening’s center (Pan et al., 2020; Pan et al., 2016). Then, the height of opening was adjusted to the participant’s shoulder height and aligned with the midline of body, and the distance of the board was adjusted to either their arm’s length or 1.3 times arm’s length, and the small table’s height to the participant’s waist height. Participants were instructed to fit the block into either a large or small opening on the custom-made board under upright stance, positioned either an arm’s length or at 1.3 times an arm’s length from the board. Thus, four different conditions were performed by participants, including close-large, close-small, far-large, and far-small conditions. Participants began with the close condition, and the size condition was randomly selected. The order was counterbalanced across participants. Moreover, the purpose of performing close conditions first was to avoid participants starting with the most difficult far-small condition. It could minimize the learning effects that affects the outcomes in the close conditions. If participants either moved their feet or if the block touched the edge of the opening during the fitting task, the trial was categorized as “failed”. For each condition, participants were required to complete five consecutive successful trails under the supervision of our experimental operator. Once a “failed” trial occurred, the participant was instructed to redo the five trials. Meanwhile, one experimental operator was required to stand in the back of the board, take the block, and put it back on the base as soon as possible. A 10-second baseline of quiet standing was recorded before the fitting task. Participants were given at least a 2 min break between conditions.

### Data analysis

The Vicon Nexus system was used to preprocess the CoP data, which was filtered with a fourth-order Butterworth low-pass filter with a cutoff frequency of 10 Hz (Rocchi, Chiari & Horak, 2002) and then exported in the format of CSV file for further analysis. Then, CoP data was divided into five trials based on the event signals from the optical sensors, where the onset of each trial was defined as the moment of rising the block and ending once the block completely passed through the opening. The standard deviation (SD) of CoP (CoP variability, SD_AP_ & SD_ML_), average CoP velocity (V_AP_ & V_ML_) at anterior-posterior (AP) and medial-lateral (ML) directions, and the 95% ellipse of sway area (SA) were calculated using the MATLAB (2021b, MathWorks, Natick, MA, USA). All these dependent variables were averaged over five trials under each condition. The higher value of these dependent variables represents worse dynamic postural stability.

To analyze the fNIRS data, the trail was defined as the first raising the block to the fifth passing the block through the opening under each condition based on the event signals from the optical sensors. The average duration of each condition was 19.74 ± 2.35 s, 24.88 ± 3.51 s, 22.45 ± 3.11 s and 30.47 ± 4.03 s in the young group, 19.77 ± 2.10 s, 28.39 ± 3.16 s, 24.24 ± 3.91 s and 33.15 ± 6.35 s in the middle-aged group, and 21.48 ± 3.47 s, 30.24 ± 7.94 s, 26.97 ± 4.47 s and 35.41 ± 8.50 s in the older group during the close-large, close-small, far-large, and far-small conditions, respectively. In the current study, only oxygenated hemoglobin (HbO_2_) data were used for further analysis. It is because that a superior signal-to-noise ratio was observed for HbO_2_than deoxygenated hemoglobin (HHb) signals (Strangman et al., 2002) and HbO_2_ and HHb were negatively associated (Cui, Bray & Reiss, 2010). The coefficients of variation (CV) for each channel of every participant were computed. Channels presenting a CV greater than 15% were excluded from subsequent data analyses, as they may include physical artifacts (*e.g.*, motion-induced instabilities of the coupling efficiency at the tissue-optical interfaces) and physiological artifacts (*e.g.*, blood-pressure-induced hemodynamics) (Pinti et al., 2019).

**Figure 1:**
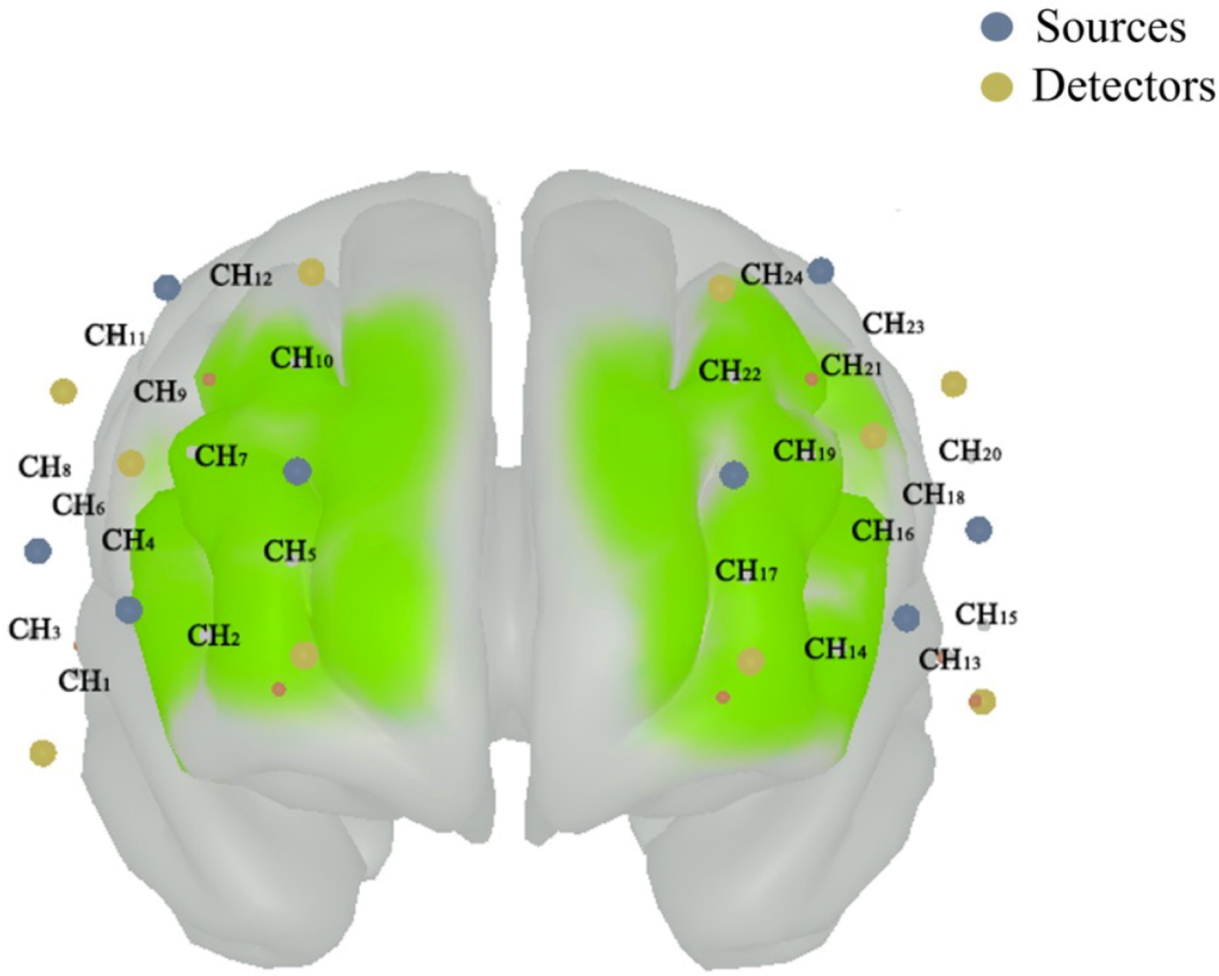
The position of composed channels by the sources (blue color) and detectors (funky yellow color) in prefrontal cortex. The right dorsolateral prefrontal cortex (PFC) includes the CH_3_, CH_6_, CH_8_, CH_9_ and CH_11_; the left dorsolateral PFC includes the CH_15_, CH_18_, CH_20_, CH_21_ and CH_23_; the right dorsomedial PFC includes the CH_2_, CH_5_, CH_7_, CH_10_ and CH_12_; the left dorsomedial PFC includes the CH_14_, CH_17_, CH_19_, CH_22_ and CH_24_; the right ventrolateral PFC includes the CH_1_ and CH_4_; and the left ventrolateral PFC includes the CH_13_ and CH_16_.

The preprocessing of the PFC activation used the Homer3 toolbox within MATLAB (The MathWorks, Inc. Natick, MA, USA), following distinct steps outlined in previous studies (Koren, Parmet & Bar-Haim, 2019; Öztürk et al., 2021): (1) converting raw data to optical density data; (2) removal of motion artifacts using the principal component analysis (tMotion = 1.0, tMask = 1.0, StdevThresh = 50 and AmpThresh = 0.5); (3) correction of motion artifacts using the spline interpolation (*p* = 0.99); (4) correction of motion artifacts using the wavelet based filter (iqr = 0.1); (5) corrected of physiological artifacts using the band-pass filter at a cutoff frequency of 0.01–0.14 Hz; (6) baseline-corrected by subtracting each trial individually from the 5 s of quiet standing; and (7) converting optical density data to concentrations. The HbO_2_ concentration signals were exported to the TXT file to further calculate. According to the channels’ positions, the average concentration of HbO_2_ on six regions of interest (Fig. 1), including right dorsolateral PFC (R_PFC_DL_), left PFC_DL_ (L_PFC_DL_), right dorsomedial PFC (R_PFC_DM_), left PFC_DM_ (L_PFC_DM_), right ventrolateral PFC (R_PFC_VL_) and left PFC_VL_ (L_PFC_VL_), were calculated.

### Statistical analysis

The two sets of dependent variables are dynamic postural stability, which includes SD_AP_, SD_ML_, V_AP_, V_ML_ and SA; and PFC activation, which includes HbO_2_concentration in the R-PFC_DL_, L-PFC_DL_, R-PFC_DM_, L-PFC_DM_, R-PFC_VL_, and L-PFC_VL_. The errors in fNIRS data are not independent across measurement channels (Huppert, 2016). Therefore, the Shapiro–Wilk’s test was only used to assess the normality of each dependent variable of dynamic postural stability (*α* = 0.05). Additionally, the multivariate normality and outliers of two sets of dependent variables were examined using Mahalanobis’ distance.

Then, two two-way repeated measure MANOVAs (between-subject factor: group & within-subject factor: condition) were performed to investigate the effects of group and condition on dynamic postural stability and PFC activation, respectively. When a repeated measure MANOVA was significant, follow-up repeat measure ANOVAs and pairwise comparisons with Bonferroni correction were employed to detect significant main effects (group and condition) and interaction effect (group × condition) for each dependent variable. Partial Eta Squared (*η*^2^*p*) was calculated for overall effects and interactions for MANOVAs and ANONAs, where *η*^2^*p* = .010 is considered a small effect size, .060 medium effect size, and .14 or higher large effect size (Cohen, 2013). Cohen’s d effect size was calculated to interpret the magnitude of specific *post hoc* comparisons, with *d* = .20 considered a small effect size, .50 medium effect size, and .80 or higher a large effect size (Cohen, 2013). The significant level was set at .05. All statistical analyses were performed using SPSS (28.0, IBM Inc., Armonk, NY, USA).

## Results

The F-test power analysis was conducted using G*Power software (Version 3.1) with repeated measures MANOVA (within-between interaction) to determine the minimal sample size. The effect size for comparing cortical activation between single-task and dual-task paradigms in older adults ranges from moderate to strong based on prior works (Pan & Zhang, 2024; Salzman et al., 2021). Therefore, we set the effect size at .65. Additionally, the analysis parameters included an alpha level of .05, a statistical power of .80, three groups, and six measurements. The results suggested a minimum sample size of 25 participants, with at least nine participants per group. In the current study, each group included seven males and eight females. Mean ± standard deviation values of demographic information and MMSE score were presented in Table 1.

**Table 1:** Mean ± standard deviation values of demographic information and Mini-Mental State Examination (MMSE) score.

	Older group	Middle-aged group	Young group
Age (years)	68.07 ± 3.58	48.73 ± 3.06	19.47 ± 0.64
Height (cm)	159.93 ± 8.43	157.80 ± 8.09	168.73 ± 6.88
Weight (kg)	60.71 ± 9.20	61.99 ± 9.99	64.77 ± 14.26
MMSE	25.33 ± 1.54	25.40 ± 1.40	28.33 ± 1.50

### Dynamic postural stability

Some dependent variables of dynamic postural stability presented non-normal distribution based on the Shapiro–Wilk’s test. These non-normally distributed dependent variables were log10 transformed before statistical analysis. Additionally, our dependent variables of the dynamic postural stability were multivariate normally distributed (MD < 20.52). Table 2 showed the Mean ± standard deviation values of dynamic postural stability among three groups under different conditions.

**Table 2:** Mean ± standard deviation values of dynamic posture stability when performing the precision fitting task among the older, middle-aged, and young groups under different conditions.

**Variables**	**Older group**	**Middle-aged group**	**Young group**
	Close-large condition		
SD_AP_^G, C & I^	34.48 ± 10.94	32.60 ± 8.83	21.89 ± 8.07
SD_ML_^G & C^	13.95 ± 8.62	8.84 ± 3.68	6.05 ± 2.96
V_AP_ (cm/s)^G, C & I^	7.45 ± 2.71	7.41 ± 2.47	5.27 ± 1.70
V_ML_(cm/s)^C & I^	3.83 ± 1.32	3.18 ± 1.28	2.66 ± 1.08
SA (cm^2^)^G & C^	39.73 ± 22.70	30.56 ± 19.99	17.10 ± 11.15
	Close-small condition		
SD_AP_^G & C^	24.12 ± 5.96	20.79 ± 4.51	20.28 ± 8.75
SD_ML_^G & C^	10.01 ± 6.52	5.98 ± 3.72	4.91 ± 2.25
V_AP_ (cm/s)^G & C^	3.84 ± 1.01	3.45 ± 0.52	3.58 ± 1.12
V_ML_(cm/s)^C^	2.35 ± 1.35	1.77 ± 0.46	2.03 ± 0.90
SA (cm^2^)^G & C^	23.34 ± 10.59	14.75 ± 7.47	13.74 ± 8.35
	Far-large condition		
SD_AP_^G, C & I^	41.95 ± 9.99	35.81 ± 9.56	29.19 ± 11.04
SD_ML_^G & C^	14.55 ± 5.51	12.23 ± 3.95	10.57 ± 3.97
V_AP_ (cm/s)^G & C^	8.99 ± 2.24	7.65 ± 2.45	7.71 ± 2.19
V_ML_(cm/s)^C^	4.53 ± 1.57	4.15 ± 1.28	4.78 ± 1.71
SA (cm^2^)^G & C^	69.53 ± 30.44	47.61 ± 20.52	39.59 ± 23.27
	Far-small condition		
SD_AP_^G, C & I^	39.15 ± 8.35	33.65 ± 12.14	24.59 ± 12.45
SD_ML_^G & C^	18.17 ± 13.65	10.29 ± 4.11	7.61 ± 3.58
V_AP_ (cm/s)^G, C & I^	6.17 ± 1.22	5.16 ± 1.41	4.31 ± 1.66
V_ML_(cm/s)^C & I^	3.83 ± 1.47	2.88 ± 0.81	2.70 ± 1.24
SA (cm^2^)^G & C^	69.25 ± 41.47	41.31 ± 26.51	26.36 ± 22.99

**Notes.**

SD_AP_ means CoP variability in the AP direction; SD_ML_ means CoP variability in the ML direction; V_AP_ means average CoP velocity in the AP direction; V_ML_ means average CoP velocity in the ML direction; and SA means sway area.

^G^ Indicates a significant group difference. ^C^ Indicated a significant condition difference. ^I^ Indicated a significant interaction difference.

**Figure 2:**
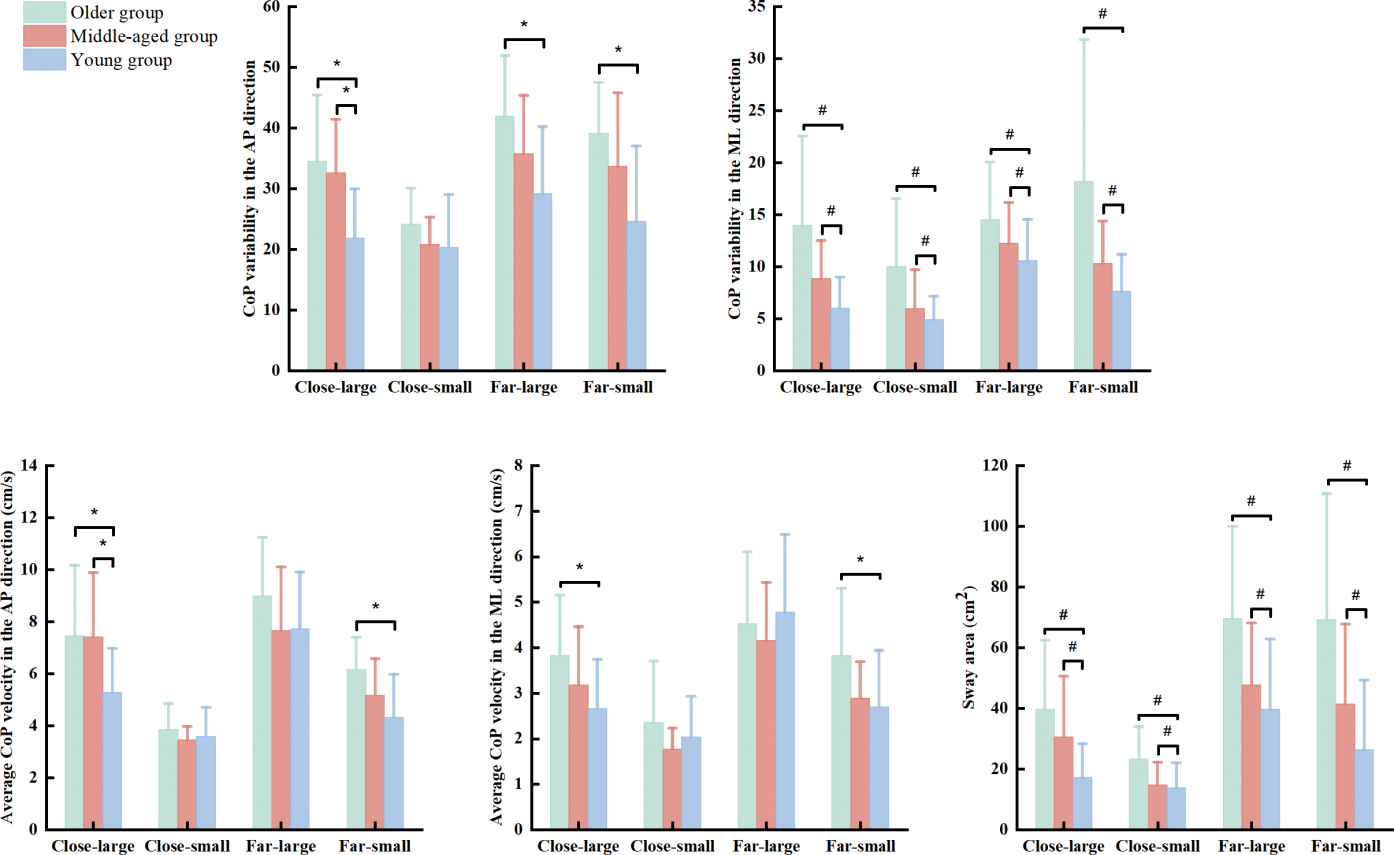
The significant group and interaction effects in the dynamic posture stability among young, middle-aged, and older group during precision fitting task. CoP means center of pressure; AP means anterior-posterior; and ML means medial-lateral. ^*^ Indicates a significant interaction difference among the groups under different conditions. ^#^ Indicates a significant difference compared to the older group regardless of the conditions.

There were significant group (Wilk’s lambda = .455, *F*(10, 76) = 3.672, *p* < .001, *η*^2^*p* = .326) and condition (Wilk’s lambda = .048, *F*(15, 28) = 36.892, *p* < .001, *η*^2^*p* = .952) effects, and group × condition interaction (Wilk’s lambda = .217, *F*(30, 56) = 2.139, *p* = .007, *η*^2^*p* = .534) effect on the association of dependent variables. Based on follow-up ANOVA with repeated measure tests, the significant effects of group, condition, and group × condition interaction were observed in the SD_AP_ (group effect: *F*(2, 42) = 8.926, *p* = .001, *η*^2^*p* = .298; condition effect: *F*(3, 126) = 42.311, *p* < .001, *η*^2^*p* = .502; & interaction effect: *F*(6, 126) = 2.819, *p* = .020, *η*^2^*p* = .118) and V_AP_ (group effect: *F*(2, 42) = 4.129, *p* = .023, *η*^2^*p* = .164; condition effect: *F*(3, 126) = 136.575, *p* < .001, *η*^2^*p* = .765; & interaction effect: *F*(6, 126) = 3.960, *p* = .002, *η*^2^*p* = .159) (Table 2). Additionally, there was significant condition effect (*F*(3, 126) = 91.357, *p* < .001, *η*^2^*p* = .952) and group × condition (*F*(6, 126) = 2.680, *p* = .021, *η*^2^*p* = .113) interaction in the V_ML_ (Table 2). *Post hoc* analysis indicated that the older group presented greater SD_AP_ than the young group in the close-large (*p* = .002 & Cohen’s *d* = 1.31), far-large (*p* = .002 & Cohen’s *d* = 1.21), and far-small (*p* < .001 & Cohen’s *d* = 1.37) conditions (Fig. 2). Also, the older group showed greater V_AP_ and V_ML_ than the young group in the close-large (V_AP_: *p* = .029 & Cohen’s *d* = 1.73 & V_ML_: *p* = .034 & Cohen’s *d* = 0.97) and far-small (V_AP_: *p* = .001 & Cohen’s *d* = 1.27& V_ML_: *p* = .021 & Cohen’s *d* = 0.83) conditions. Additionally, the middle-aged group showed grater SD_AP_ (*p* = .005 & Cohen’s *d* = .77) and V_AP_ (*p* = .029 & Cohen’s *d* = .77) compared to the young group in the close-large condition (Fig. 2).

The ANOVA with repeated measure tests further observed group and condition effects in the SD_ML_ (group effect: *F*(2, 42) = 10.919, *p* < .001, *η*^2^*p* = .342; & condition effect: *F*(3, 126) = 27.039, *p* < .001, *η*^2^*p* = .392) and SA (group effect: *F*(2, 42) = 13.014, *p* < .001, *η*^2^*p* = .383; & condition effect: *F*(3, 126) = 58.553, *p* < .001, *η*^2^*p* = .582). *Post hoc* test reported that the older group showed greater SD_ML_ (young group: *p* < 0.001 & Cohen’s *d* = .96 & middle-age group: *p* = 0.028 & Cohen’s *d* = .66) and SA (young group: *p* < 0.001 & Cohen’s *d* = .94 & middle-age group: *p* = 0.030 & Cohen’s *d* = .58) than the young and middle-aged groups across the conditions (Fig. 2). We did not report the condition effect since our interests were the effects of group and interaction between group and condition.

### Prefrontal cortex activation

Our dependent variables of the PFC activation were multivariate normally distributed within each group of the independent variables (MD < 22.46). Table 3 showed the mean ± standard deviation values of HbO_2_ concentration in the PFC among three groups under different conditions.

**Table 3:** Mean ± standard deviation values of HbO 2 (µm/ml) in the prefrontal cortex when performing the precision fitting task among the older, middle-aged, and young groups under different conditions.

**Variables**	**Older group**	**Middle-aged group**	**Young group**
	Close-large condition		
R_PFC_DL_^G & C^	0.28 ± 0.23	0.15 ± 0.22	0.042 ± 0.18
L_PFC_DL_^G^	0.28 ± 0.20	0.082 ± 0.15	0.036 ± 0.24
R_PFC_DM_^G & C^	0.24 ± 0.23	0.10 ± 0.22	−0.011 ± 0.19
L_PFC_DM_^G & C^	0.22 ± 0.18	0.10 ± 0.18	0.025 ± 0.26
R_PFC_VL_^G & C^	0.35 ± 0.28	0.29 ± 0.34	0.048 ± 0.33
L_PFC_VL_^G & C^	0.31 ± 0.29	0.14 ± 0.19	0.023 ± 0.32
	Close-small condition		
R_PFC_DL_^G & C^	0.51 ± 0.33	0.33 ± 0.61	0.10 ± 0.19
L_PFC_DL_^G^	0.45 ± 0.32	0.27 ± 0.52	0.048 ± 0.17
R_PFC_DM_^G & C^	0.42 ± 0.31	0.25 ± 0.60	0.15 ± 0.19
L_PFC_DM_^G & C^	0.42 ± 0.31	0.32 ± 0.55	−0.056 ± 0.30
R_PFC_VL_^G & C^	0.65 ± 0.43	0.47 ± 0.95	0.19 ± 0.31
L_PFC_VL_^G & C^	0.60 ± 0.43	0.37 ± 0.56	0.068 ± 0.23
	Far-large condition		
R_PFC_DL_^G & C^	0.53 ± 0.57	0.23 ± 0.32	0.14 ± 0.39
L_PFC_DL_^G^	0.53 ± 0.53	0.12 ± 0.36	0.061 ± 0.27
R_PFC_DM_^G & C^	0.51 ± 0.57	0.21 ± 0.36	0.12 ± 0.34
L_PFC_DM_^G & C^	0.48 ± 0.52	0.22 ± 0.39	−0.066 ± 0.34
R_PFC_VL_^G & C^	0.73 ± 0.64	0.38 ± 0.55	0.18 ± 0.50
L_PFC_VL_^G & C^	0.61 ± 0.59	0.27 ± 0.43	−0.071 ± 0.64
	Far-small condition		
R_PFC_DL_^G & C^	0.54 ± 0.33	0.49 ± 0.30	0.35 ± 0.42
L_PFC_DL_^G^	0.47 ± 0.41	0.25 ± 0.29	0.17 ± 0.31
R_PFC_DM_^G & C^	0.54 ± 0.29	0.48 ± 0.33	0.27 ± 0.46
L_PFC_DM_^G & C^	0.68 ± 0.41	0.42 ± 0.39	0.14 ± 0.43
R_PFC_VL_^G & C^	0.84 ± 0.48	0.75 ± 0.57	0.57 ± 0.64
L_PFC_VL_^G & C^	0.96 ± 0.53	0.62 ± 0.58	0.37 ± 0.60

**Notes.**

R means right; L means left; PFC_DL_ means dorsolateral prefrontal cortex; PFC_DM_ means dorsomedial prefrontal cortex; PFC_VL_ means ventrolateral prefrontal cortex.

^G^ Indicates a significant group difference. ^C^ Indicated a significant condition difference. ^I^ Indicated a significant interaction difference.

**Figure 3:**
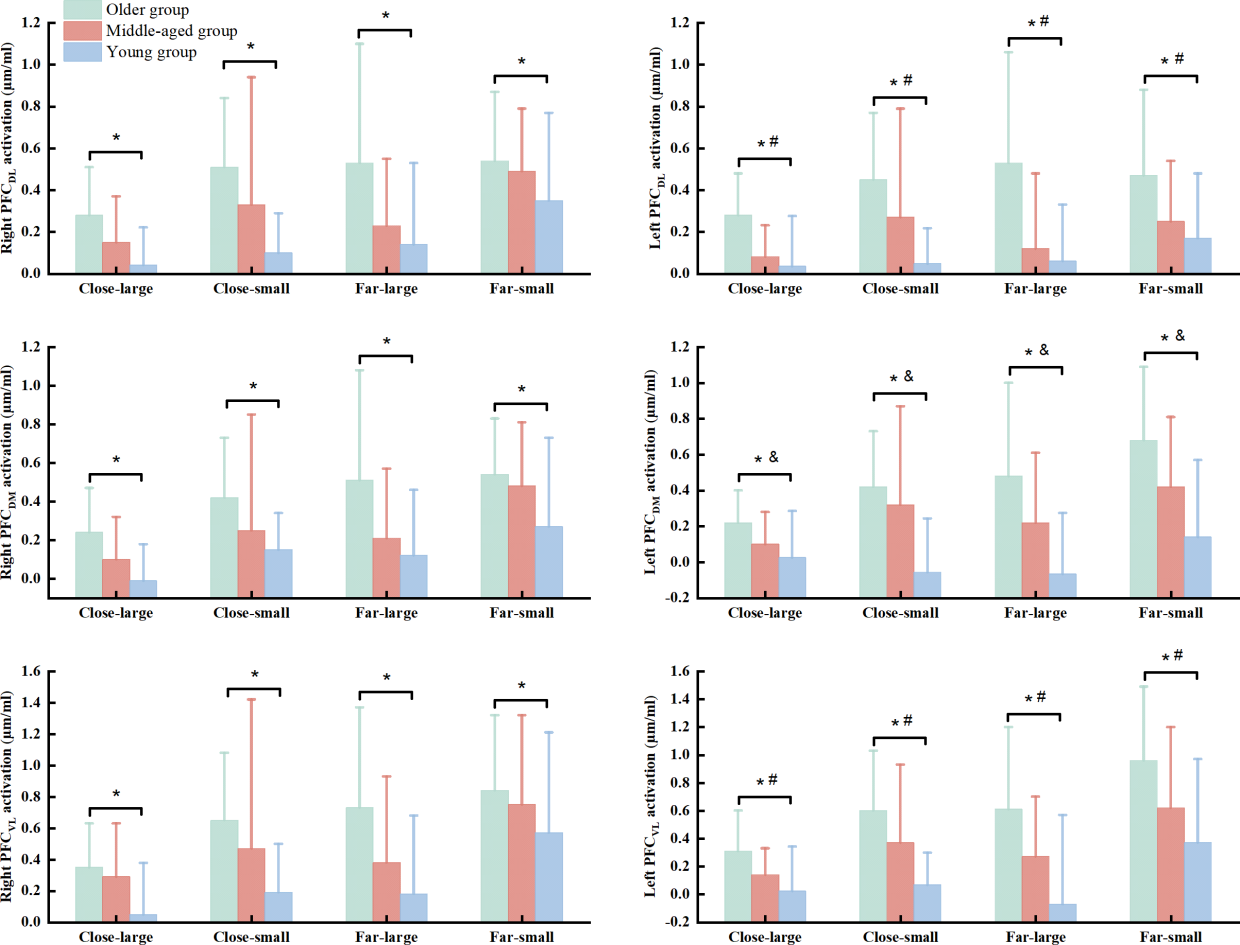
The significant group effect in the cortical activation among young, middled-aged, and older group during precision fitting task. PFC_DL_ means dorsolateral prefrontal cortex; PFC_DM_ means dorsomedial prefrontal cortex; PFC_VL_ means ventrolateral prefrontal cortex. ^*^ Indicates a significant difference between the older and young groups regardless of the conditions. ^#^ Indicates a significant difference between the older and middle-aged groups regardless of the conditions. ^&^ Indicates a significant difference between the middle-aged and young groups regardless of the conditions.

There were significant group (Wilk’s lambda = .467, *F*(12, 74) = 3.455, *p* = .003, *η*^2^*p* = .317) and condition (Wilk’s lambda = .295, *F*(18, 25) = 3.327, *p* = .003, *η*^2^*p* = .75) effects on the association of dependent variables. No significant group × condition interaction effect (Wilk’s lambda = .253, *F*(36, 50) = 1.371, *p* = .150, *η*^2^*p* = .497) was observed in the MANOVA model. Follow-up ANOVA with repeated measure tests reported group effect in the R_PFC_DL_ (*F*(2, 42) = 7.693, *p* = .001, *η*^2^*p* = .268), L_PFC_DL_ (*F*(2, 42) = 11.076, *p* < .001, *η*^2^*p* = .345), R_PFC_DM_ (*F*(2, 42) = 7.701, *p* = .001, *η*^2^*p* = .268), L_PFC_DM_ (*F*(2, 42) = 15.637, *p* < .001, *η*^2^*p* = .427), R_PFC_VL_ (*F*(2, 42) = 6.920, *p* = .003, *η*^2^*p* = .248), and L_PFC_VL_ (*F*(2, 42) = 12.325, *p* < .001, *η*^2^*p* = .370) and condition effect in the R_PFC_DL_ (*F*(3, 126) = 5.933, *p* = .002, *η*^2^*p* = .124), R_PFC_DM_ (*F*(3, 126) = 6.390, *p* < .001, *η*^2^*p* = .132), L_PFC_DM_ (*F*(3, 126) = 5.675, *p* = .001, *η*^2^*p* = .119), R_PFC_VL_ (*F*(3, 126) = 6.935, *p* = .001, *η*^2^*p* = .142), and L_PFC_VL_ (*F*(3, 126) = 10.674, *p* < .001, *η*^2^*p* = .203) (Table 3). *Post hoc* test indicated that the older group presented greater HbO_2_ activation in the R_PFC_DL_ (*p* = .001 & Cohen’s *d* = .85), L_PFC_DL_ (*p* < .001 & Cohen’s *d* = 1.08), R_PFC_DM_ (*p* = .001 & Cohen’s *d* = .84), L_PFC_DM_ (*p* < .001 & Cohen’s *d* = 1.18), R_PFC_VL_ (*p* = .002 & Cohen’s *d* = .79), and L_PFC_VL_ (*p* < .001 & Cohen’s *d* = 1.04) than the young group, while older group also showed greater HbO_2_ concentration in the L_PFC_DL_ (*p* = .007 & Cohen’s *d* = .38) and L_PFC_VL_ (*p* = .041 & Cohen’s *d* = .54) than the middled-aged group across the conditions (Fig. 3). Also, the middle-aged group presented greater HbO_2_ concentration in the L_PFC_DM_ (*p* = .008 & Cohen’s *d* = .36) than the young group (Fig. 3). We did not report the condition effect since our interests were the group and interaction effects among group and between group and condition.

## Discussion

The current study aimed to explore the effects of precision fitting task on the dynamic posture stability and PFC activation at six different subregions among the young, middle-aged, and old groups. Our results indicated that (1) the older group presented worse dynamic postural stability compared to the young group in all of the conditions, except for the close-small condition; (2) the middle-age group only showed worse dynamic postural stability compared to the young group in the close-large condition; (3) regardless the conditions, the middle-aged group exhibited better dynamic postural stability compared to the older group; (4) the older group presented greater HbO_2_ concentration in all PFC’s subregions compared to the young group; and (5) the middle-age group showed lower HbO_2_ concentration in the L_PFC_DL_ and L_PFC_VL_ compared to the older group, but they had greater HbO_2_ concentration in the L_PFC_DM_ compared to the young group. Our observations are consistent with our hypothesis.

The young group presented better dynamic postural stability compared to the middle-aged and older groups in the close-large condition. The observation is consistent with prior works (Huang & Brown, 2013; McNevin, Weir & Quinn, 2013; Walz et al., 2023). For instance, one study indicated that the gait speed in the timed up & go task with goal-directed arm-movement task presented faster in the young group compared to the middle-aged and older groups (Walz et al., 2023). The possible explanation is a decline in controlling whole-body postural fluctuations with aging when the upright stance is perturbated by a precision goal-directed task (Haddad et al., 2013). Compared to close-large condition, the fitting task is becoming more difficult when decreasing the opening size (enhancing the fitting precision) and increasing the opening distance (enhancing the postural constraint) (Coats et al., 2016; Huang & Brown, 2013; Potocanac & Duysens, 2017; Sarlegna, 2006). To successfully complete these conditions, participants need to control body configurations to the limits of upright stability and to gear upper body actions to locate the object simultaneously (Haddad et al., 2013; Ornkloo & Von Hofsten, 2007). Increased task difficulty leads to an increase in completion time, less smooth of arm trajectory, worse endpoint accuracy, and long-latency response to arm movement (Coats et al., 2016; Huang & Brown, 2013; Potocanac & Duysens, 2017; Sarlegna, 2006). Interestingly, the current study did not observe the significant difference in dynamic postural stability between the young and middle-aged groups in the close-small, far-large, and far-small conditions. The middle-aged group presented better dynamic postural stability than older adults across the conditions. An explanation is that the study protocol is fixed order from close to far conditions. Middle-aged adults could have the ability to learn fitting skills from close conditions, because previous study indicated that motor learning was significantly slower in adults over 62 years when learning novel visuomotor task (Smith et al., 2005). For the older adults, they presented worse dynamic postural stability than young adults in the far-large and far-small conditions. Previous work indicated that far conditions demand an increased degree of coordination between posture and manual motor task compared to close conditions (Rossi, Mitnitski & Feldman, 2002). Sensory systems are required to additionally process to update the position of trunk, hand and opening to optimize movement accuracy at far conditions (Cheng et al., 2012; Goodman et al., 2018). Thus, it might be the deficits in the capacity of using the existed visuospatial information or online visual control to guide the fitting task in older adults that induced the increased dynamic postural instability (Cheng et al., 2012; Grabowski & Mason, 2014). Another possible explanation is that older adults show decreasing the complexities of multi-joint movement *via* the strategy of freezing the trunk and arm, but is not allowed to suppress dynamic postural fluctuations and to speed up fitting movement among older adults (Fuster & Bressler, 2015; Gaveau et al., 2014; Haddad et al., 2012).

The current study also observed that older group had greater HbO_2_ activation in the PFC_DL_, PFC_DM_, and PFC_VL_ than young adults across conditions. Prior works have demonstrated that heightened HbO_2_activation in the PFC subregions (PFC_DL_, PFC_DM_, PFC_VL_) in response to the goal-directed behaviors may be associated with processing visuospatial, visuomotor, and visual object information, respectively (Yamagata et al., 2012; Brovelli et al., 2017). The precision fitting task requires the sensory feedback from hand and target position and adapting postural configurations to optimize movement accuracy due to increasing the terminal accuracy (Lowrey, Nashed & Scott, 2017; Sarlegna, 2006; Zhou et al., 2011). Older adults have shown the impaired ability of processing the visuospatial and visuomotor information in goal-directed tasks (Cheng et al., 2012; Grabowski & Mason, 2014). Accordingly, we speculate that additional attention-demanding executive control resources are used to monitor the interaction among environment, arm and trunk movement, target, and upright stance in older adults. In addition, the middle-aged group presented greater HbO_2_activation in the PFC_DM_ than young group across the conditions. Considering the function of increased PFC_DM_, middle-aged adults may require substantial effort in integrating sensory input and motor planning for precise coordination in goal-directed tasks compared to young adults (Brovelli et al., 2017). These observations may suggest that middle-aged adults initially degenerate the ability in processing the visuomotor information, rather than visuospatial and visual object information (Yamagata et al., 2012; Brovelli et al., 2017). This suggestion is further supported by other results in the current study, which indicates smaller HbO_2_ activation in the PFC_DL_ and PFC_VL_ in the middle-aged group compared to older group.

Taken the dynamic postural stability and HbO_2_ activation in the PFC regions together, this study provide evidence indicating that loss of automaticity in coordination task-dependent postural control may emerges earlier in adulthood at midlife (Potocanac & Duysens, 2017; Sarlegna, 2006). It is because we observed greater HbO_2_ activation in the PFC_DM_ and worse dynamic posture stability in middle-aged adults compared to young adults. In some contexts, heightened PFC activation in middle-aged adults is utilized to preserve dynamic postural stability compared to young adults in the close-small, close-far, and far-small conditions. Hence, middle-aged adults can be explained by the “compensation” hypothesis, which presents slight decline in their brain function and cognition (Fettrow et al., 2021; Levin et al., 2014). The older group presented greater HbO_2_ activation in all the PFC subregions and worse dynamic postural stability compared to middle-aged and young groups. These observations might be supported by the “de-differentiation” hypothesis (Levin et al., 2014). Meanwhile, increased HbO_2_ activation in the PFC regions fails to improve dynamic postural stability in older adults due to less specificity of PFC functions (Fettrow et al., 2021). These observations may imply the importance of neural processing at the highest levels of the control hierarchy in coordination dynamic postural control in goal-directed tasks. This is clinically important because it links the potential mechanism in loss of automatic coordination of dynamic postural control with aging and might be a strong predictor of risk of falls. Moreover, the degeneration of postural automaticity in middle-aged adults should not be overlooked. Future studies can focus on identifying rehabilitation protocols that boost the ability in mediating task planning and execution for cognitive and motor functions in both middle-aged and older groups.

This is the first study to simultaneously investigate PFC activation and dynamic postural stability in three different age groups (young, middle-aged, and older adults) when performing precision fitting task. However, the limitations of this study should not be overlooked. First, this study was not a randomized controlled trial. It may have introduced selection bias and affected our results. Second, the sample size is small, which limits the generalizability of the observations. Third, although fNIRS has good temporal specificity, it can only record cortical activation and restricting the region of interest to the PFC in this study. This prevents us from analyzing deeper regions such as subcortical structures as well as other higher-order cognitive regions such as the motor cortex.

## Conclusion

In the current study, the dynamic postural stability presented young group > middle-aged group > older group, which suggested that individuals reaching to middle-age is associated with an impaired ability in suppressing dynamic postural fluctuations during the precision fitting task. Additionally, middle-aged adults presented higher HbO_2_ activation in the PFC_DM_ than young adults, as well as showed lower HbO_2_ activation in the PFC_DL_ and PFC_VL_ than older adults across the conditions. This observation may further suggest that individuals reaching middle-age are associated with an impaired ability in processing the visuomotor information during the goal-directed tasks. These observations are clinically important because they suggested that rehabilitation interventions improving the visuomotor-related function could improve the dynamic postural control and minimize the risk of falls.

##  Supplemental Information

10.7717/peerj.18548/supp-1 Data S1 Raw data

## Additional Information and Declarations

### Data Availability

The following information was supplied regarding data availability: The raw measurements are available in the Supplementary File.

## References

[ref-1] Anderson BA (2018). Controlled information processing, automaticity, and the burden of proof. Psychonomic Bulletin & Review.

[ref-2] Bouisset S, Do M-C (2008). Posture, dynamic stability, and voluntary movement. Neurophysiologie Clinique/Clinical Neurophysiology.

[ref-3] Brovelli A, Badier J-M, Bonini F, Bartolomei F, Coulon O, Auzias G (2017). Dynamic reconfiguration of visuomotor-related functional connectivity networks. The Journal of Neuroscience.

[ref-4] Cheng KC, McKay SM, King EC, Maki BE (2012). Does aging impair the capacity to use stored visuospatial information or online visual control to guide reach-to-grasp reactions evoked by unpredictable balance perturbation?. The Journals of Gerontology: Series A.

[ref-5] Claassen JA, Levine BD, Zhang R (2009). Dynamic cerebral autoregulation during repeated squat-stand maneuvers. Journal of Applied Physiology.

[ref-6] Clark DJ (2015). Automaticity of walking: functional significance, mechanisms, measurement and rehabilitation strategies. Frontiers in Human Neuroscience.

[ref-7] Clark MC, Czaja SJ, Weber RA (1990). Older adults and daily living task profiles. Human Factors.

[ref-8] Coats RO, Fath AJ, Astill SL, Wann JP (2016). Eye and hand movement strategies in older adults during a complex reaching task. Experimental Brain Research.

[ref-9] Cohen J (2013). Statistical power analysis for the behavioral sciences.

[ref-10] Cui X, Bray S, Reiss AL (2010). Functional near infrared spectroscopy (NIRS) signal improvement based on negative correlation between oxygenated and deoxygenated hemoglobin dynamics. Neuroimage.

[ref-11] Cui GH, Yao YH, Xu RF, Tang HD, Jiang GX, Wang Y, Wang G, Chen SD, Cheng Q (2011). Cognitive impairment using education-based cutoff points for CMMSE scores in elderly Chinese people of agricultural and rural Shanghai China. Acta Neurologica Scandinavica.

[ref-12] Duncan A, Meek JH, Clemence M, Elwell CE, Fallon P, Tyszczuk L, Cope M, Delpy DT (1996). Measurement of cranial optical path length as a function of age using phase resolved near infrared spectroscopy. Pediatric Research.

[ref-13] Era P, Sainio P, Koskinen S, Haavisto P, Vaara M, Aromaa A (2006). Postural balance in a random sample of 7,979 subjects aged 30 years and over. Gerontology.

[ref-14] Fettrow T, Hupfeld K, Tays G, Clark DJ, Reuter-Lorenz PA, Seidler RD (2021). Brain activity during walking in older adults: implications for compensatory *versus* dysfunctional accounts. Neurobiology of Aging.

[ref-15] Funahashi S, Andreau JM (2013). Prefrontal cortex and neural mechanisms of executive function. Journal of Physiology-Paris.

[ref-16] Fuster JM, Bressler SL (2015). Past makes future: role of pFC in prediction. Journal of Cognitive Neuroscience.

[ref-17] Galgon AK, Shewokis PA, Tucker CA (2010). Changes in standing postural control during acquisition of a sequential reaching task. Gait & Posture.

[ref-18] Gaveau V, Pisella L, Priot A-E, Fukui T, Rossetti Y, Pélisson D, Prablanc C (2014). Automatic online control of motor adjustments in reaching and grasping. Neuropsychologia.

[ref-19] Glover S, Wall MB, Smith AT (2012). Distinct cortical networks support the planning and online control of reaching-to-grasp in humans. European Journal of Neuroscience.

[ref-20] Goodman R, Crainic VA, Bested SR, Wijeyaratnam DO, De Grosbois J, Tremblay L (2018). Amending ongoing upper-limb reaches: visual and proprioceptive contributions?. Multisensory Research.

[ref-21] Goodman R, Tremblay L (2021). Older adults rely on somatosensory information from the effector limb in the planning of discrete movements to somatosensory cues. Experimental Gerontology.

[ref-22] Grabowski PJ, Mason AH (2014). Age differences in the control of a precision reach to grasp task within a desktop virtual environment. International Journal of Human-Computer Studies.

[ref-23] Haaland KY, Harrington DL, Grice JW (1993). Effects of aging on planning and implementing arm movements. Psychology and Aging.

[ref-24] Haddad JM, Claxton LJ, Keen R, Berthier NE, Riccio GE, Hamill J, Van Emmerik RE (2012). Development of the coordination between posture and manual control. Journal of Experimental Child Psychology.

[ref-25] Haddad JM, Rietdyk S, Claxton LJ, Huber JE (2013). Task-dependent postural control throughout the lifespan. Exercise and Sport Sciences Reviews.

[ref-26] Huang M-H, Brown SH (2013). Age differences in the control of postural stability during reaching tasks. Gait & Posture.

[ref-27] Huang MH, Brown SH (2015). Effects of task context during standing reach on postural control in young and older adults: a pilot study. Gait & Posture.

[ref-28] Huppert TJ (2016). Commentary on the statistical properties of noise and its implication on general linear models in functional near-infrared spectroscopy. Neurophotonics.

[ref-29] Kaller CP, Rahm B, Spreer J, Weiller C, Unterrainer JM (2011). Dissociable contributions of left and right dorsolateral prefrontal cortex in planning. Cerebral Cortex.

[ref-30] Klaassen EB, Plukaard S, Evers EA, De Groot RH, Backes WH, Veltman DJ, Jolles J (2016). Young and middle-aged schoolteachers differ in the neural correlates of memory encoding and cognitive fatigue: a functional MRI study. Frontiers in Human Neuroscience.

[ref-31] Koren Y, Parmet Y, Bar-Haim S (2019). Treading on the unknown increases prefrontal activity: a pilot fNIRS study. Gait & Posture.

[ref-32] Kwon D, Maillet D, Pasvanis S, Ankudowich E, Grady CL, Rajah MN (2016). Context memory decline in middle aged adults is related to changes in prefrontal cortex function. Cerebral Cortex.

[ref-33] Leonard JA, Gritsenko V, Ouckama R, Stapley PJ (2011). Postural adjustments for online corrections of arm movements in standing humans. Journal of Neurophysiology.

[ref-34] Levin O, Fujiyama H, Boisgontier MP, Swinnen SP, Summers JJ (2014). Aging and motor inhibition: a converging perspective provided by brain stimulation and imaging approaches. Neuroscience & Biobehavioral Reviews.

[ref-35] Lowrey CR, Nashed JY, Scott SH (2017). Rapid and flexible whole body postural responses are evoked from perturbations to the upper limb during goal-directed reaching. Journal of Neurophysiology.

[ref-36] Mansouri FA, Tanaka K, Buckley MJ (2009). Conflict-induced behavioural adjustment: a clue to the executive functions of the prefrontal cortex. Nature Reviews Neuroscience.

[ref-37] McNevin N, Weir P, Quinn T (2013). Effects of attentional focus and age on suprapostural task performance and postural control. Research Quarterly for Exercise and Sport.

[ref-38] Mederos S, Sanchez-Puelles C, Esparza J, Valero M, Ponomarenko A, Perea G (2021). GABAergic signaling to astrocytes in the prefrontal cortex sustains goal-directed behaviors. Nature Neuroscience.

[ref-39] Ornkloo H, Von Hofsten C (2007). Fitting objects into holes: on the development of spatial cognition skills. Developmental Psychology.

[ref-40] Öztürk Ö, Algun ZC, Bombacı H, Erdoğan SB (2021). Changes in prefrontal cortex activation with exercise in knee osteoarthritis patients with chronic pain: an fNIRS study. Journal of Clinical Neuroscience.

[ref-41] Pan J, Liu C, Li L, Zhang S (2020). The effect of Tai Chi exercise on postural time-to-contact in manual fitting task among older adults. Gait & Posture.

[ref-42] Pan J, Liu C, Zhang S, Li L (2016). Tai Chi can improve postural stability as measured by resistance to perturbation related to upper limb movement among healthy older adults. Evidence-based Complementary & Alternative Medicine.

[ref-43] Pan J, Zhang S (2024). Dual-task effect on center of pressure oscillations and prefrontal cortex activation between young and older adults. Research Quarterly for Exercise and Sport.

[ref-44] Peters R (2006). Ageing and the brain. Postgraduate Medical Journal.

[ref-45] Pinti P, Scholkmann F, Hamilton A, Burgess P, Tachtsidis I (2019). Current status and issues regarding pre-processing of fNIRS neuroimaging data: an investigation of diverse signal filtering methods within a general linear model framework. Frontiers in Human Neuroscience.

[ref-46] Potocanac Z, Duysens J (2017). Online adjustments of leg movements in healthy young and old. Experimental Brain Research.

[ref-47] Rocchi L, Chiari L, Horak F (2002). Effects of deep brain stimulation and levodopa on postural sway in Parkinson’s disease. Journal of Neurology, Neurosurgery, and Psychiatry.

[ref-48] Rossi E, Mitnitski A, Feldman AG (2002). Sequential control signals determine arm and trunk contributions to hand transport during reaching in humans. Physiology Journals.

[ref-49] Salzman T, Vallejo DTobón, Polskaia N, Michaud L, St-Amant G, Lajoie Y, Fraser S (2021). Hemodynamic and behavioral changes in older adults during cognitively demanding dual tasks. Brain and Behavior.

[ref-50] Sarlegna FR (2006). Impairment of online control of reaching movements with aging: a double-step study. Neuroscience Letters.

[ref-51] Schöner G, Bildheim L, Zhang L (2024). Toward a neural theory of goal-directed reaching movements. Progress in motor control.

[ref-52] Smith C, Walton A, Loveland A, Umberger G, Kryscio R, Gash D (2005). Memories that last in old age: motor skill learning and memory preservation. Neurobiology of Aging.

[ref-53] Strangman G, Culver JP, Thompson JH, Boas DA (2002). A quantitative comparison of simultaneous BOLD fMRI and NIRS recordings during functional brain activation. Neuroimage.

[ref-54] Tedeschi R (2023). Assessment of postural control and proprioception using the delos postural proprioceptive system. Reabilitacijos mokslai: slauga, kineziterapija, ergoterapija.

[ref-55] Walz ID, Waibel S, Kuhner A, Gollhofer A, Maurer C (2023). Age-related changes in mobility assessments correlate with repetitive goal-directed arm-movement performance. BMC Geriatrics.

[ref-56] Yamagata T, Nakayama Y, Tanji J, Hoshi E (2012). Distinct information representation and processing for goal-directed behavior in the dorsolateral and ventrolateral prefrontal cortex and the dorsal premotor cortex. The Journal of Neuroscience.

[ref-57] Zhou S-S, Fan J, Lee TM, Wang C-Q, Wang K (2011). Age-related differences in attentional networks of alerting and executive control in young, middle-aged, and older Chinese adults. Brain and Cognition.

